# Distinguishing Focal Cortical Dysplasia From Glioneuronal Tumors in Patients With Epilepsy by Machine Learning

**DOI:** 10.3389/fneur.2020.548305

**Published:** 2020-11-24

**Authors:** Yi Guo, Yushan Liu, Wenjie Ming, Zhongjin Wang, Junming Zhu, Yang Chen, Lijun Yao, Meiping Ding, Chunhong Shen

**Affiliations:** ^1^Department of General Practice, School of Medicine, Second Affiliated Hospital, Zhejiang University, Hangzhou, China; ^2^Epilepsy Center, School of Medicine, Second Affiliated Hospital, Zhejiang University, Hangzhou, China; ^3^School of Computer Science, Fudan University, Shanghai, China; ^4^Department of Neurology, School of Medicine, Second Affiliated Hospital, Zhejiang University, Hangzhou, China; ^5^Department of Neurosurgery, School of Medicine, Second Affiliated Hospital, Zhejiang University, Hangzhou, China; ^6^Shanghai Pudong New Area Mental Health Center, Tongji University School of Medicine, Shanghai, China

**Keywords:** machine learning, epilepsy, focal cortical dysplasia, glioneuronal tumors, distinguish

## Abstract

**Purpose:** We are aiming to build a supervised machine learning-based classifier, in order to preoperatively distinguish focal cortical dysplasia (FCD) from glioneuronal tumors (GNTs) in patients with epilepsy.

**Methods:** This retrospective study was comprised of 96 patients who underwent epilepsy surgery, with the final neuropathologic diagnosis of either an FCD or GNTs. Seven classical machine learning algorithms (i.e., Random Forest, SVM, Decision Tree, Logistic Regression, XGBoost, LightGBM, and CatBoost) were employed and trained by our dataset to get the classification model. Ten features [i.e., Gender, Past history, Age at seizure onset, Course of disease, Seizure type, Seizure frequency, Scalp EEG biomarkers, MRI features, Lesion location, Number of antiepileptic drug (AEDs)] were analyzed in our study.

**Results:** We enrolled 56 patients with FCD and 40 patients with GNTs, which included 29 with gangliogliomas (GGs) and 11 with dysembryoplasic neuroepithelial tumors (DNTs). Our study demonstrated that the Random Forest-based machine learning model offered the best predictive performance on distinguishing the diagnosis of FCD from GNTs, with an F1-score of 0.9180 and AUC value of 0.9340. Furthermore, the most discriminative factor between FCD and GNTs was the feature “age at seizure onset” with the Chi-square value of 1,213.0, suggesting that patients who had a younger age at seizure onset were more likely to be diagnosed as FCD.

**Conclusion:** The Random Forest-based machine learning classifier can accurately differentiate FCD from GNTs in patients with epilepsy before surgery. This might lead to improved clinician confidence in appropriate surgical planning and treatment outcomes.

## Introduction

Focal cortical dysplasia (FCD) is a distinctive malformation of cortical development that is highly associated with refractory epilepsy. Around 12–40% of patients with FCD were submitted to surgery for refractory epilepsy (1). Neuropathological findings have also reported glioneuronal tumors (GNTs) are another important cause of refractory epilepsy in children and young adults, including gangliogliomas (GGs) and dysembryoplastic neuroepithelial tumors (DNTs) (2). Previous studies have demonstrated that patients with FCD and GNTs have different postoperative seizure outcomes. Up to 80% patients with GNTs could achieve seizure-free during the follow-up. However, only 40–50% patients with FCD experienced no seizures after surgery (3, 4). As for the surgical protocol in many patients with FCD, wide cortical resection over the MRI-delineated lesion with invasive electroencephalography is frequently recommended, due to obscure histologic boundary and poor prognosis (5). In contrast, recent studies on tumor-associated epilepsy have emphasized that total surgical resection of the tumor is sufficient and effective for seizure control in most patients with GNTs (6). Thus, it is crucial to make the differential diagnosis of FCD and GNTs preoperatively. However, their clinical manifestation and imaging findings could be similar, especially in cases of mass-like FCD (7). What' more, type III FCD was accompanied by an additional brain lesion as noted in the classification system by the International League Against Epilepsy (ILAE) (8).

Some factors have been reported to differentiate FCD from GNTs before surgery. Rácz's et al. study indicated that age at epilepsy onset was younger in patients with FCD compared to those with GNTs (9). Though ^18^F-FDG PET can't contribute to the differentiation of FCD and GNTs, ^11^C-methionine PET identified a significant difference between them (10). But, up to now, ^11^C-methionine PET was unavailable in most hospitals worldwide. Several surface EEG biomarkers were also revealed to be significantly correlated with an underlying cortical dysplasia (11). Despite these factors, it remained a challenge to effectively differentiate FCD from GNTs preoperatively.

Machine learning, as an important branch of artificial intelligence, has been applied to automated seizure detection (12), prediction of antiepileptic drugs (AEDs) response (13), pre-surgical planning and surgical outcome prediction (14). In the present study, we adopted supervised machine learning-based algorithms to train the classifier to differentiate FCD from GNTs, using seven representative classification algorithms, i.e., Decision Tree (15), Random Forest (16), Logistic Regression (17), Support Vector Machine (SVM) (18), XGBoost (19), Catboost (20), and LightGBM (21). In addition, we included several features to predict different pathological results, attempting to identify the most valuable feature. Based on our results, one can use the trained classifier to make a diagnosis prediction of FCD or GNTs before surgery, hence helping clinicians to make better surgical planning.

## Methods

### Patients and Definitions

The study was retrospectively conducted from January 2013 to December 2018 in the Second Affiliated Hospital of Zhejiang University, a tertiary referral hospital in Zhejiang, China. All the patients underwent epilepsy surgery at the Epilepsy Center. Inclusion criteria were as follows: (1) patients were diagnosed as epilepsy according to the guidelines for the Classification and Diagnosis of Epilepsy of ILAE (22). (2) the neuropathologic diagnosis of either FCD or GNTs was established by two senior neuropathologists (8, 23), discrepancies were discussed and resolved by verification from a third senior neuropathologist. (3) all the patients underwent a non-invasive pre-surgical evaluation, including long-term video-EEG monitoring, high-resolution MRI with epilepsy sequence and PET-CT for some of them; for patients whose surgical protocols were with difficulties, invasive evaluation with the stereo-electroencephalography was carried out. Among 308 patients who underwent epilepsy surgery from January 2013 to December 2018 in our center, 98 patients met inclusion criteria, 2 patients did not obtain informed consent, and 96 patients were included in the final analysis. Informed consents were obtained from all the participants, and the study was approved by the Second Affiliated hospital of Zhejiang University School of Medicine Ethics Committee.

The information of ten features (i.e., Gender, Past history, Age at seizure onset, Course of disease, Seizure type, Seizure frequency, Scalp EEG biomarkers, MRI features, Lesion location, Number of AEDs) were recorded. Past history included encephalitis, perinatal brain injury, febrile convulsion, traumatic brain injuries and other known secondary causes. Seizure type was classified according to the new operational classification by ILAE (24), and seizure frequency was grouped into four categories: every few years, once a year, once few months and several times a month (25). Video EEG (VEEG) was performed using digital VEEG systems (Nicolet, VIASYS, United States and Biologic, NATUS, United States), with scalp electrodes placed according to the international 10–20 system. All the patients were monitored for at least 24 h. For the patients with long-term monitoring, the first 24 h recordings were chosen without AEDs tapering. Two EEG experts were blind to the MRI results and underlying histopathology. EEG recordings were evaluated in both referential and bipolar montages, and positive biomarkers were considered to be present when consensus between two independent EEG experts was achieved. The positive biomarkers of FCD were defined as the presence of continuous epileptiform discharges, two types of rhythmic epileptiform discharges, polyspikes, repetitive activity and polyspikes, frequent rhythmic bursting epileptiform activity, or repetitive discharges according to Epitashvili's et al. study (11). Examples of positive EEG biomarkers were shown in (Supplementary Figure 1). As for MRI protocols, patients were conducted on a 3.0T scanner (MR750, GE Healthcare, United States) with an 8-channel brain phased array coil. High resolution coronal T2-weighted images perpendicular to the long axis of the hippocampus were acquired using spoiled gradient echo sequence with TR/TE = 5,518/176 ms, flip angle = 110°, slice thickness = 2 mm, matrix = 512 × 512. Sagittal 3D T1-weighted images were acquired using brain volume imaging (BRAVO) sequence with TR/TE = 8.2/3.2 ms, TI = 450 ms, flip angle = 12°, slice thickness = 1 mm, matrix = 256 × 256. High resolution axial T2-weighted images and fluid-attenuated inversion-recovery (FLAIR) sequence were also obtained. Contrast-enhanced images T1-and T2-weighted images were obtained, if necessary. Typical MRI characteristics of FCD type I included subtle white matter signal changes and regional reduction of the white matter volume. Typical MRI characteristics of FCD type II included focal cortical thickening, blurring of the gray-white matter interface, focally increased signal on T2-weighted imaging, and a tail of increased signal from the cortex to an underlying ventricle on T2-weighted imaging (transmantle sign) (26, 27). GGs usually presents as a cyst with an enhancing mural nodule, with minimal to no surrounding edema and no significant mass effect. Foci of calcification are frequent (40–50%) in GGs and areas of cortical dysplasia can be seen adjacent to the tumor (28, 29). On MRI, DNTs appear well-demarcated and frequently wedge shaped, hypointense on T1WI, and hyperintense on T2WI, lack of edema and mass effect. Calcifications can be seen in 20% DNTs, and 20% DNTs have nodular or ring-like enhancement (28, 29). Typical characteristics of GGs and DNTs on MRI imaging were considered as typical characteristics of GNTs (28, 29). Typical MRI characteristics of FCD or GNTs were considered when consensus between two independent neuroradiologists was achieved. Examples of MRI for FCD and GNTs were shown in (Supplementary Figure 2).

### Machine Learning

Our work was aiming to build a binary classification model capable of distinguishing FCD from GNTs. The process of the supervised machine learning-based analysis included the following steps, i.e., data preprocessing, feature selection, algorithm selection, parameter tuning, and performance evaluation. The method was as the same as our previous study (13). The workflow of data preparation and machine-learning based modeling was shown in the (Supplementary Figure 3).

#### Data Preprocessing

In our analysis, 56 patients with FCD and 40 with GNTs were recruited. To solve the unbalanced sample problem, we over-sampled the minority type to 56 by using the SMOTE technique (30). (https://www.jair.org/index.php/jair/article/view/10302). Then we randomly split the entire dataset into a training and validation dataset and a test dataset. The training and validation dataset were used to train and validate the prediction model, while the test dataset was applied to evaluate the prediction performance of the trained model. We used 50% of patients for training and validation, the rest for test. The aim of the training and validation stage is to find an optimal set of parameters that can achieve the highest prediction performance. We further applied the 5-fold cross-validation method by randomly dividing the training and validation dataset into 5 subsets with equal sample sizes. The cross-validation process was repeated for 5 rounds. For each round, one of the 5 subsets were retained as the validation data to evaluate the model, and the remaining 4 subsets were used for training. We have made our dataset available to the public via Harvard Dataverse (https://dataverse.harvard.edu/dataset.xhtml?persistentId=doi:10.7910/DVN/6F7QPP).

#### Algorithm Selection and Parameter Tuning

For machine learning algorithm selection, we included classical algorithms such as Random Forest, SVM, Decision Tree and Logistic Regression, as well as new algorithms, i.e., XGBoost, LightGBM, and CatBoost. For each algorithm, we should determine an optimal set of parameters. Based on the training and validation dataset, we applied grid search to go through the parameter space, which covers a finite set of parameter combinations. For each parameter combination, we evaluated the model's prediction performance using the training and validation dataset. We record the parameters leading to the highest F1-score. To train and evaluate the classification model (31), we used the scikit-learn library, a representative open source machine learning toolkit, written in the Python programming language. This library supports a number of supervised machine learning algorithms, including Decision Tree, Random Forest, Logistic Regression, Support Vector Machine (SVM), XGBoost, Catboost, and LightGBM. After selecting a specified algorithm, the scikit-learn library is able to process the training and validation dataset to obtain a classification model. Then this model can be further applied to the test dataset.

#### Performance Evaluation

Based on the test dataset, we used precision, recall, F1-score, and the AUC (Area Under the ROC Curve) value to evaluate the predictive performance of our trained model (32). Precision was the fraction of patients with FCD who were finally diagnosed with FCD. Recall was the fraction of patients with FCD who have been adequately identified by the model. F1-score was the harmonic mean of precision and recall, with its best value at 1 and worst value at 0. F1-score was calculated as follows:

$$\begin{array}{l} \text{F}1=\frac{2\cdot precision\cdot recall}{precision+recall} \end{array}$$

From the perspective of clinicians, high precision means that our prediction rarely over-reports or over-represents the fraction of patients with predicted FCD who are in fact diagnosed with FCD. Meanwhile, high recall means the fraction of patients with FCD who are uncovered accurately. A higher value of F1-score indicates a better overall predictive performance of a classifier. AUC is another important metric for evaluating a classification model's performance, which denotes the probability that a machine learning algorithm will rank higher of a random positive instance than a randomly chosen negative instance. The value of AUC is between 0 and 1. For a perfect classifier, the AUC value will be 1. For a completely random classifier, the AUC value will be 0.5. If the AUC value is smaller than 0.5, we could invert all the outputs of the classifier and obtain a new AUC value larger than 0.5. An AUC value close to 1 indicates that the model is good at distinguishing FCD from GNTs.

### Statistical Analysis

Statistical analysis was performed using python. Continuous variable (course of disease) with normal distribution was represented as mean ± standard deviation (SD), non-normal variable (age at seizure onset) was reported as median [interquartile range (IQR)]. Categorical variables were described in the form of frequency and percentage. Independent student's *t*-test were conducted to compare the means of the continuous variables with normal distribution while Welch's *t*-test was used if the data was not normally distributed. Chi-Squared (χ^2^) Statistics was used to compare the frequencies of categorical variables between FCD and GNTs Groups. And we calculated the Chi-Square (χ^2^) Statistics to evaluate the dependence of each selected feature on different pathological results (33). A larger χ^2^ value indicated a better discriminative power of the feature. A value of *p* < 0.05 was considered significant. All the tests were two tailed.

## Results

### Patient Characteristics

**Figure 1:**
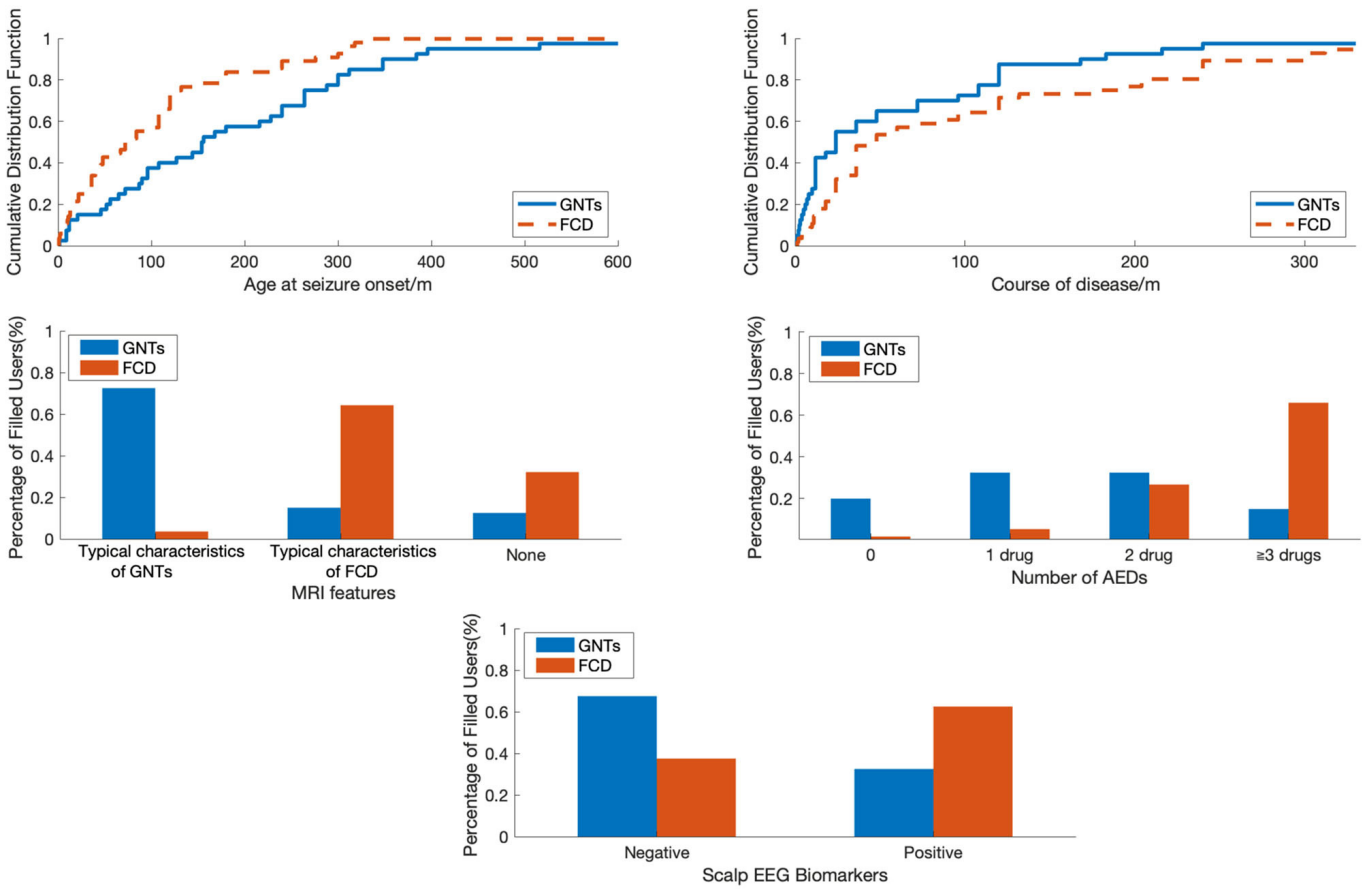
The comparison of patients with FCD and GNTs in terms of different features. FCD, focal cortical dysplasia; GNTs, glioneuronal tumors; AEDs, antiepileptic drugs.

A total of 96 patients who underwent epilepsy surgery were analyzed in our study, including 56 patients with FCD (FCD I: *n* = 16; FCD II: *n* = 40) and 40 patients with GNTs (GG: *n* = 29; DNTs = 11). Ten features were reviewed and recorded; the details were shown in Table 1. The median age at seizure onset (months) in FCD group was much lower than that in GNTs group (77 vs. 155, *P* = 0.002, also see Figure 1); Course of disease (months) in FCD group was longer than that in GNTs group, but not statistically significant (105 vs. 69, *P* = 0.12, also see Figure 1). Thirty-five (62.5%) patients with FCD showed scalp EEG biomarkers of FCD, whereas only 13 (32.5%) patients with GNTs had the positive biomarkers (*p* = 0.04, also see Figure 1). Thirty six (64.3%) patients with FCD had typical MRI characteristics of FCD, and 29 (72.5%) patients in GNTs group had typical MRI characteristics of GNTs (*p* < 0.001, also see Figure 1). As for AEDs, 37 (66.0%) patients in FCD group were taking more than 3 kinds of AEDs, while only 6 (15.0%) patients in GNTs group were taking 3 or more kinds of AEDs (*p* < 0.001, also see Figure 1). However, there were no significant differences in gender, past history, seizure type, seizure frequency, and lesion location between two groups with FCD and GNTs.

**Table 1:** Clinical characteristics of included patients with FCD and GNTs.

**Variable**	**FCD (*n* = 56)**	**GNTs (*n* = 40)**	**Overall (*n* = 96)**	***P*-value**
Gender, *n* (%)				0.593
Female	27 (48.2%)	16 (40%)	43 (44.8%)	
Male	29 (51.8%)	24 (60%)	53 (55.2%)	
Past History, *n* (%)				0.949
Negative	52 (92.9%)	37 (92.5%)	89 (92.7%)	
Positive	4 (7.1%)	3 (7.5%)	7 (7.3%)	
Age at seizure onset (months), median (IQR)	77 (31, 125)	155 (70, 270)	108 (36, 228)	0.002^*^
Course of disease (months), Mean ± SD	105 ± 113	69 ± 107	90 ± 111	0.12
Seizure type, *n* (%)				0.978
FAS	14 (17.5%)	10 (18.2%)	24 (17.7%)	
FIAS	40 (50%)	22 (40%)	62 (45.9%)	
FBTCS	26 (32.5%)	23 (41.8%)	49 (36.4%)	
Seizure frequency, *n* (%)				0.184
Every few years	2 (3.6%)	7 (17.5%)	9 (9.4%)	
Once a year	0 (0%)	1 (2.5%)	1 (1.0%)	
Once few months	4 (7.1%)	5 (12.5%)	9 (9.4%)	
Several times a month	50 (89.3%)	27 (67.5%)	77 (80.2%)	
Scalp EEG biomarkers of FCD, *n* (%)				0.040^*^
Negative	21 (37.5%)	27 (67.5%)	48 (50.0%)	
Positive	35 (62.5%)	13 (32.5%)	48 (50.0%)	
MRI features, *n* (%)				<0.001^*^
Typical characteristics of GNTs	2 (3.6%)	29 (72.5%)	31 (32.3%)	
Typical characteristics of FCD	36 (64.3%)	6 (15%)	42 (43.8%)	
None	18 (32.1%)	5 (12.5%)	23 (23.9%)	
Lesion location, *n* (%)				0.130
Frontal lobe	31 (55.4%)	3 (7.5%)	34 (35.4%)	
Temporal lobe	19 (33.9%)	34 (85%)	53 (55.2%)	
Parietal lobe	4 (7.1%)	2 (5%)	6 (6.3%)	
Occipital lobe	1 (1.8%)	1 (2.5%)	2 (2.1%)	
Insular lobe	1 (1.8%)	0 (0%)	1(1.0%)	
Number of AEDs, *n* (%)				<0.001^*^
None	1 (1.8%)	8 (20%)	9 (9.4%)	
1 drug	3 (5.4%)	13 (32.5%)	16 (16.7%)	
2 drugs	15 (26.8%)	13 (32.5%)	28 (29.1%)	
≧3 drugs	37 (66.0%)	6 (15.0%)	43 (44.8%)	

FCD, focal cortical dysplasia; GNTs, glioneuronal tumors; FAS, focal aware seizure; FIAS, focal impaired awareness seizure; FBTCS, focal to bilateral tonic-clonic seizure; AEDs, antiepileptic drugs; IQR, interquartile range; SD, standard deviation;

* *P < 0.05 was considered statistically significant*.

### Machine Learning Algorithms Used to Distinguish FCD From GNTs

With the current dataset, we adopted supervised machine learning algorithms to preoperatively predict pathological diagnosis of patients with epilepsy. A wide variety of machine learning algorithms were selected to build classification models, including Random Forest, Catboost, SVM, XGBoost, LightGBM, Logistic Regression, and Decision Tree. As shown in Table 2, the F1-scores of these seven models (Random Forest, Catboost, SVM, XGBoost, LightGBM, Logistic Regression, and Decision Tree) were 0.9180, 0.9000, 0.8621, 0.8667, 0.8750, 0.8889, and 0.8000, and the AUC values were 0.9340, 0.9515, 0.9055, 0.9630, 0.8531, 0.9132, and 0.7873, respectively. The precision/positive predictive value and recall/sensitivity for each model were also described in Table 2. Overall, our data revealed that Random Forest and Catboost were most effective in distinguishing patients with FCDs from those with GNTs. Furthermore, Random Forest-based classifier achieved the highest F1-score of 0.9180 and an AUC value of 0.9340, providing the best discriminatory ability in the prediction of pathological diagnosis.

**Table 2:** The performance of different algorithms to distinguish FCD from GNTs.

**Algorithm**	**Precision/ Positive predictive value**	**Recall/ Sensitivity**	**F1-Score**	**AUC**
Random forest	0.8750	0.9655	0.9180	0.9340
Catboost	0.8710	0.9310	0.9000	0.9515
Logistic regression	0.9600	0.8276	0.8889	0.9132
LightGBM	0.8000	0.9655	0.8750	0.8531
XGBoost	0.8387	0.8966	0.8667	0.9630
SVM	0.8621	0.8621	0.8621	0.9055
Decision tree	0.7742	0.8276	0.8000	0.7873

*FCD, focal cortical dysplasia; GNTs, glioneuronal tumors*.

### The Features Used to Distinguish FCD From GNTs

Next, the Chi-Square analysis was employed to identify the discriminative power of each feature to preoperatively make the diagnosis of FCD or GNTs. The top 5 ranked features that effectively contributed to distinguishing FCD from GNTs were Age at seizure onset, Course of disease, MRI features, Number of AEDs, and Scalp EEG biomarkers, with the Chi-square values of 1,213.000, 334.800, 19.969, 13.946, and 4.200, respectively (Table 3).

**Table 3:** The discriminative power of different features to distinguish FCD from GNTs.

**Rank**	**Feature**	**Chi-square value**
1	Age at seizure onset	1,213.000
2	Course of disease	334.800
3	MRI features	19.969
4	Number of AEDs	13.946
5	Scalp EEG biomarkers	4.200
6	Lesion location	2.287
7	Seizure frequency	1.760
8	Gender	0.285
9	Past history	0.004
10	Seizure type	0.001

*FCD, focal cortical dysplasia; GNTs, glioneuronal tumors; AEDs, antiepileptic drugs*.

To visualize the difference between patients with FCD and GNTs, we analyzed in terms of Age at seizure onset, Course of disease, MRI features, Number of AEDs and Scalp EEG biomarkers as shown in Figure 1. Age at seizure onset was revealed to be the most discriminative feature to distinguish between patients with FCD and GNTs, meaning that younger age at seizure onset would increase the probability of the diagnosis of FCD.

## Discussion

In the present study, we demonstrated that the Random Forest-based machine learning model provided the best predictive performance on distinguishing FCD from GNTs, with an F1-score of 0.9180 and AUC value of 0.9340. Of ten included features, “Age at seizure onset” was revealed to be the most discriminative feature. With this supervised machine learning-based approach, one would accurately differentiate FCD from GNTs in patients with epilepsy before surgery, allowing clinicians to make the surgical planning properly and individually.

For all the patients who underwent epilepsy surgery, the ultimate desired outcomes were complete seizure freedom without further AEDs. Therefore, accurate preoperative diagnosis of FCD or GNTs based upon clinical features was of great importance, when planning the extent of resection and choosing the invasive evaluation as noted above. With widespread use in image recognition, language processing, and data mining, machine learning-based techniques have received increasing attention in medical applications, including the use of epilepsy (14). One challenge is that there are a series of potential supervised ML algorithms which could be selected. To our knowledge, which algorithm is the most suitable one for our problem is unknown. Our study focused on the differential diagnosis of FCD and GNTs before surgery, indicating that two classification algorithms (Random Forest and Catboost) were quite effective to predict between FCD and GNTs. Particularly, the Random Forest-based model performed best in prediction. Logistic regression was a widely used statistical method with an F1-score of 0.8889 in our study, which was much lower compared to that of Random Forest. Consequently, our Random Forest-based model would be considered as a potential and powerful classifier to predict the preoperative pathological diagnosis for patients with epilepsy. Consistent with our result, Paldino et al. have indicated that the Random Forest classifier achieved 100% sensitivity and 95.4% specificity in predicting language impairment with DTI-based whole-brain tractography data from pediatric patients with malformations of cortical development (34). A later study conducted by Grinspan et al. has also demonstrated that the Random Forest classifier achieved AUCs of 84.1 and 73.4% at each center in predicting emergency department visit rates for the following year, using a combination of demographic characteristics, insurance, comorbidity, and medication data in medical records at two pediatric referral centers (35). In our study, the consistent rate between conventional preoperative diagnosis and postoperative pathology was 76%, while the consistent rate was 89.6% when preoperative Random Forest algorithm was used to predict postoperative pathology, showing a statistically significant difference (Supplementary Table 1, χ^2^ = 6.184, *p* = 0.013). As far as we know, this was the first study reporting that machine learning-based algorithms could be used to differentiate FCD from GNTs in patients with epilepsy. For the next step, we will use a larger sample to train our algorithm. One practical challenge is that different hospitals might host their patient databases on computers with different operating systems, including Windows, Linux and MacOSX. Our algorithm is implemented using the scikit-learn library (https://scikit-learn.org/stable/), which is an open source library written in the Python programming language. Thanks to the cross-platform nature of Python, our algorithm can be directly deployed on computers with any mainstream operating system without modification. Our algorithm could directly access a hospital's database of patient records, and read the patient information automatically to provide the predicted diagnosis of FCD or GNTs. In short, our algorithm has no special requirement for either the operating system or the computer hardware. It is convenient to be employed in clinical applications. If the diagnosis given by the classifier is FCD, wider cortical resection over the MRI-delineated lesion may be taken into consideration by neurosurgeons, in order to achieve favorable seizure outcomes. Furthermore, having a good knowledge of the potential postsurgical outcome may improve clinicians' and patients' confidence in epilepsy surgery.

As for the top 5 ranked features which contributed most to distinguishing FCD from GNTs in patients with epilepsy, the feature “Age at seizure onset” had the highest Chi-square value at 1,213.000, suggesting patients who have the younger age at seizure onset were more likely to be diagnosed as FCD finally. This result was consistent with the study from Rácz et al. which indicated that age at epilepsy onset was significantly earlier in patients with FCD than that in GNTs (9). The second feature “Course of disease” had the Chi-square value at 334.800, suggesting that epileptic patients with FCD had a longer course of disease compared to patients with GNTs. A possible explanation could be that GNTs group had a higher proportion (72.5%) of patients with typical characteristics of GNTs and consequently underwent surgical treatment earlier, which was also a reason for the number difference of AEDs between two groups. As the commonly used method to distinguish FCD from GNTs, “MRI” was the third feature with the Chi-square value at 19.969, which was however obviously lower than the former. Epitashvili et al. have demonstrated that six surface EEG biomarkers (continuous epileptiform discharges, two types of rhythmic epileptiform discharges, polyspikes, repetitive activity, and polyspikes, frequent rhythmic bursting epileptiform activity or repetitive discharges) were significantly associated with an underlying cortical dysplasia (11). However, the single feature “Scalp EEG biomarkers” was also shown with less significance in our study, meaning the requirement of machine learning-based comprehensive evaluation progressed from signal processing analyses.

The predictive performance of a model depends on the large scale of dataset, the number and quality of features, and the design of the algorithms. Our study had some limitations. First, the current dataset was collected at a local tertiary hospital, and the sample may not be representative of all the regions in China and other countries. In the future, a prospective multicenter study with a larger sample size should be required. Second, ten features were included in our study, however the weight of each feature in the final model differed, which possibly increased the risk of overfitting or bias. Finally, some features were not included in this work, such as multiple seizure types, other MRI sequences (DTI) and PET-CT finding. The diagnostic validity of machine learning-based approach was associated with comprehensive parameters, thereby more features were considered, the higher level of performance we would achieve.

## Conclusion

Taken together, this study highlighted the potential of a supervised machine learning-based model to differentiate FCD from GNTs in patients with epilepsy before surgery, contributing to appropriate surgical planning. With the availability and convenience of this model, clinicians will benefit from the novel approach in clinical applications.

## Data Availability Statement

The raw data supporting the conclusions of this article will be made available by the authors, without undue reservation.

## Ethics Statement

The studies involving human participants were reviewed and approved by the Second Affiliated hospital of Zhejiang University School of Medicine Ethics Committee. Written informed consent to participate in this study was provided by the participants' legal guardian/next of kin.

## Author Contributions

YG and CHS: conceptualization and writing. YSL, LJY, and YC: methodology. WJM, ZJW, and JMZ: data collecting and confirmation. MPD and CHS: project administration and supervision. All authors contributed to the article and approved the submitted version.

## Conflict of Interest

The authors declare that the research was conducted in the absence of any commercial or financial relationships that could be construed as a potential conflict of interest.
