# Supplementary material for: Distinguishing Focal Cortical Dysplasia From Glioneuronal Tumors in Patients With Epilepsy by Machine Learning
Source: Front Neurol. 2020 Nov 24;11:548305. doi: 10.3389/fneur.2020.548305 (PMC7732488; doi:10.3389/fneur.2020.548305)
Supplement: Supplementary file 1 [file Table_1.DOCX]

**Supplementary Figure 1** **Examples of positive EEG biomarkers for focal cortical dysplasia (FCD)**


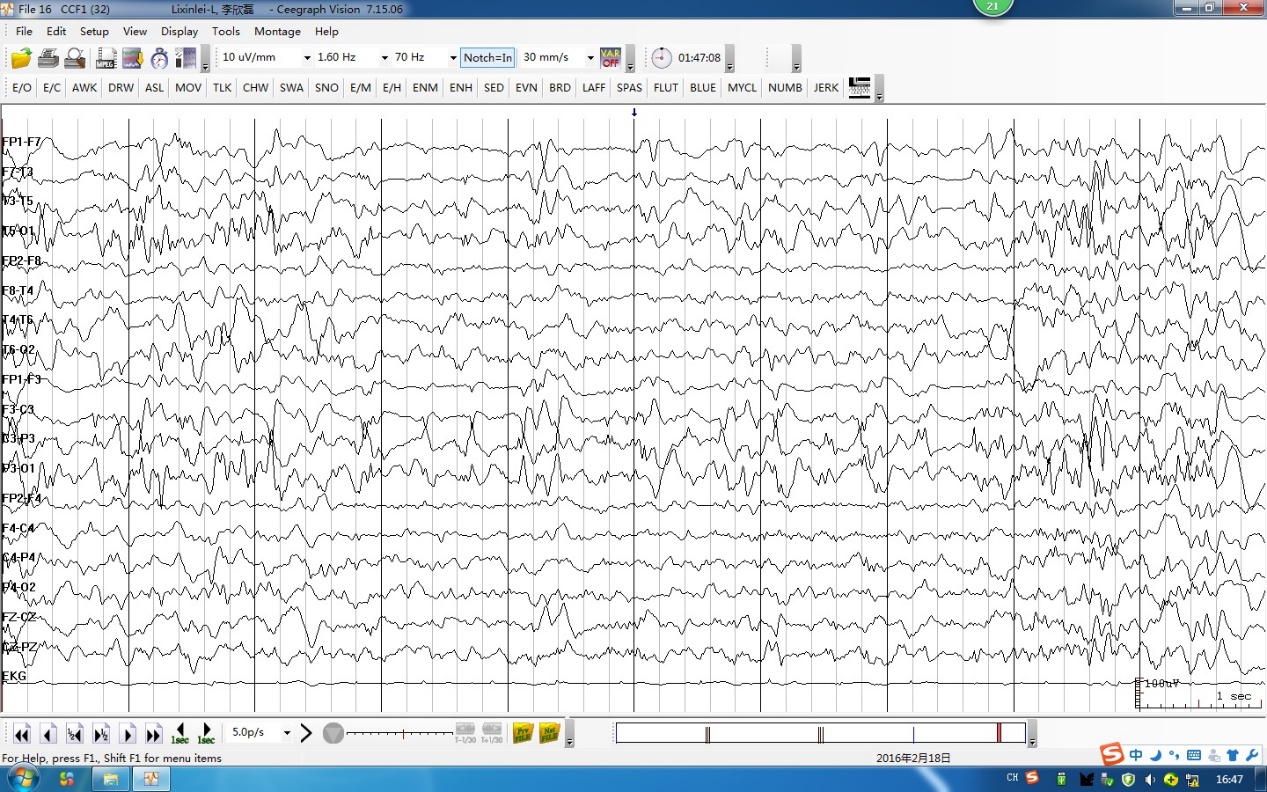
 Supplementary Fig.1a. Example of continuous epileptiform discharges.


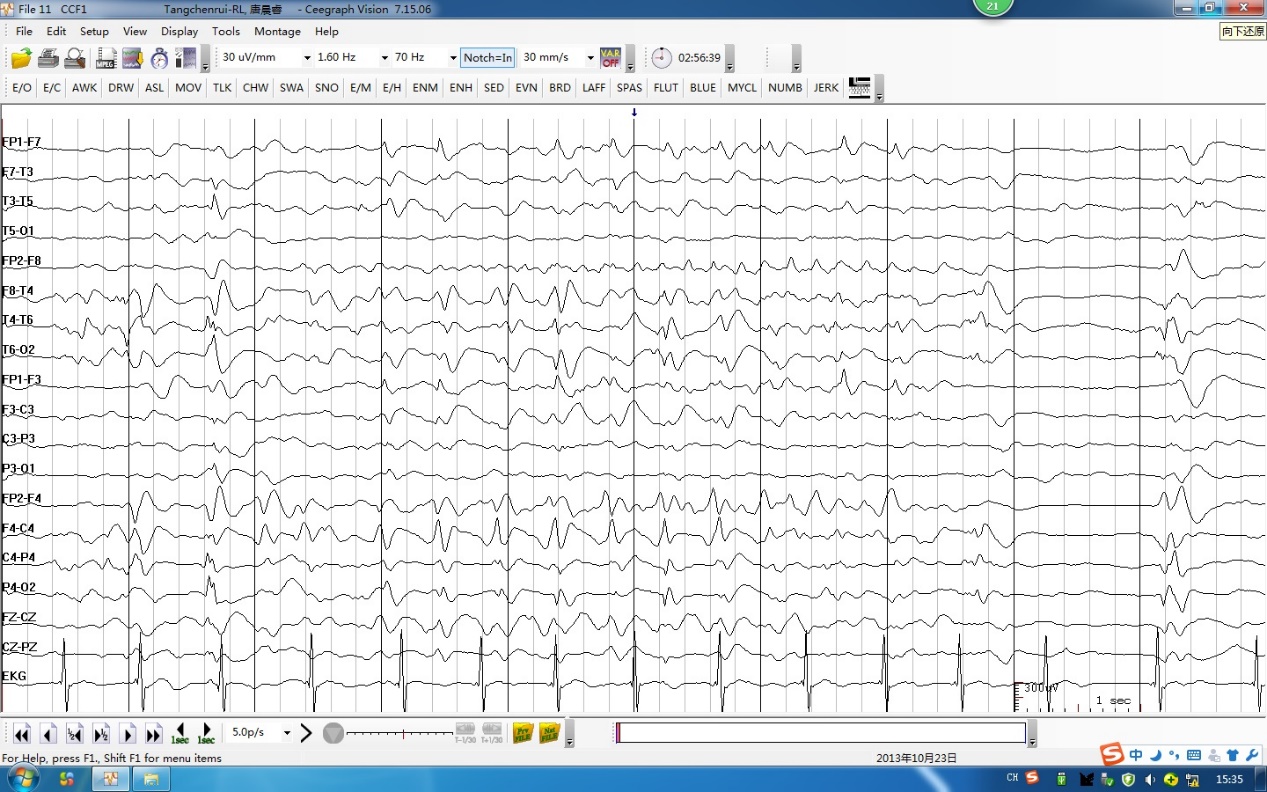


Supplementary Fig.1b. Example of two types of rhythmic epileptiform discharges


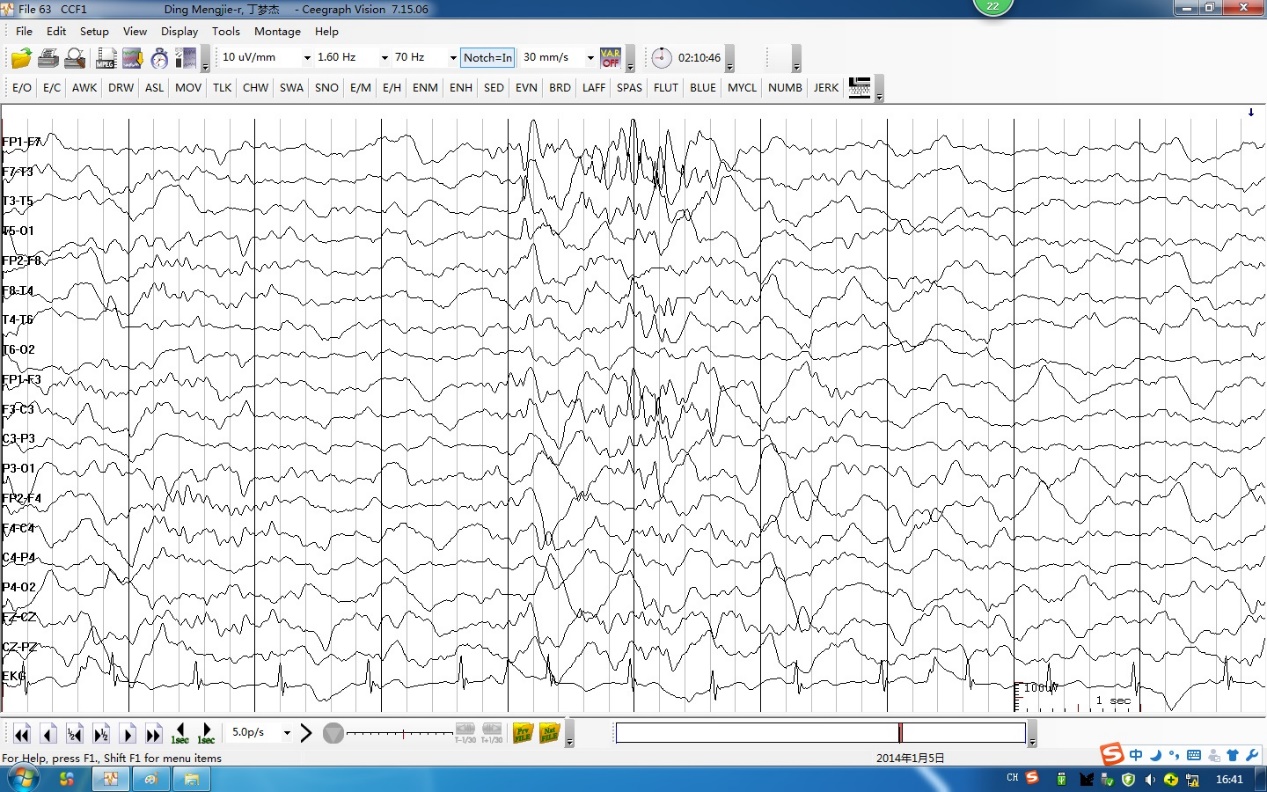


Supplementary Fig.1c. Example of polyspikes.


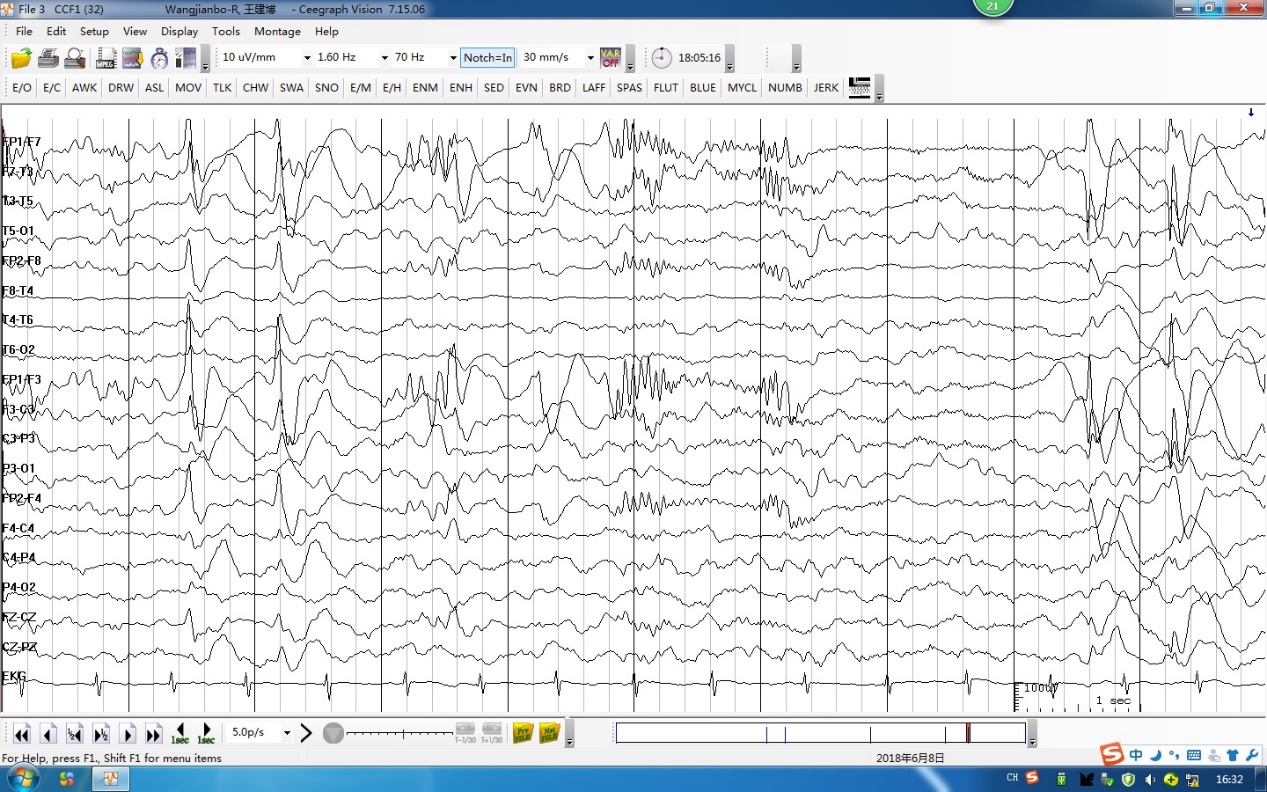
 Supplementary Fig.1d. Example of repetitive activity and polyspikes.


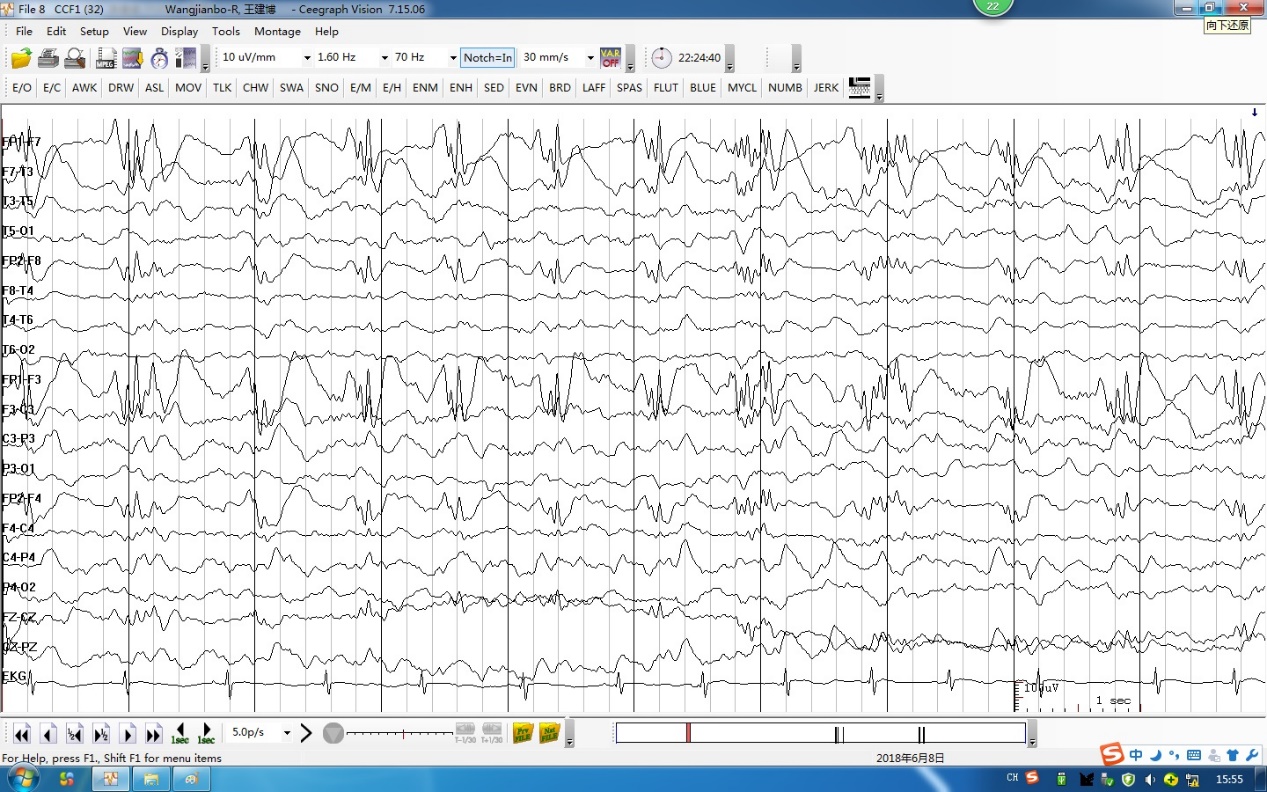
 Supplementary Fig.1e. Example of frequent rhythmic bursting epileptiform activity


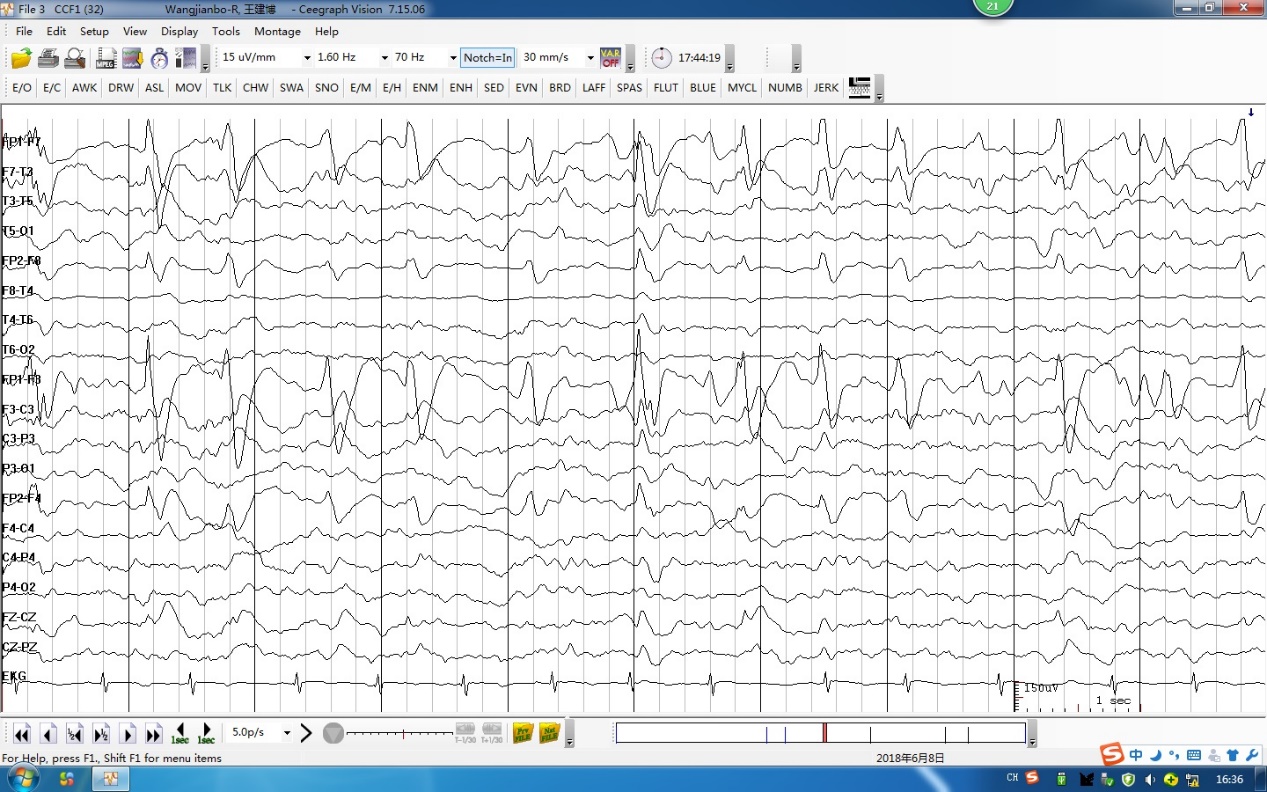
 Supplementary Fig.1f. Example of repetitive discharges

**Supplementary figure 2 Examples of MRI for FCD and GNTs**


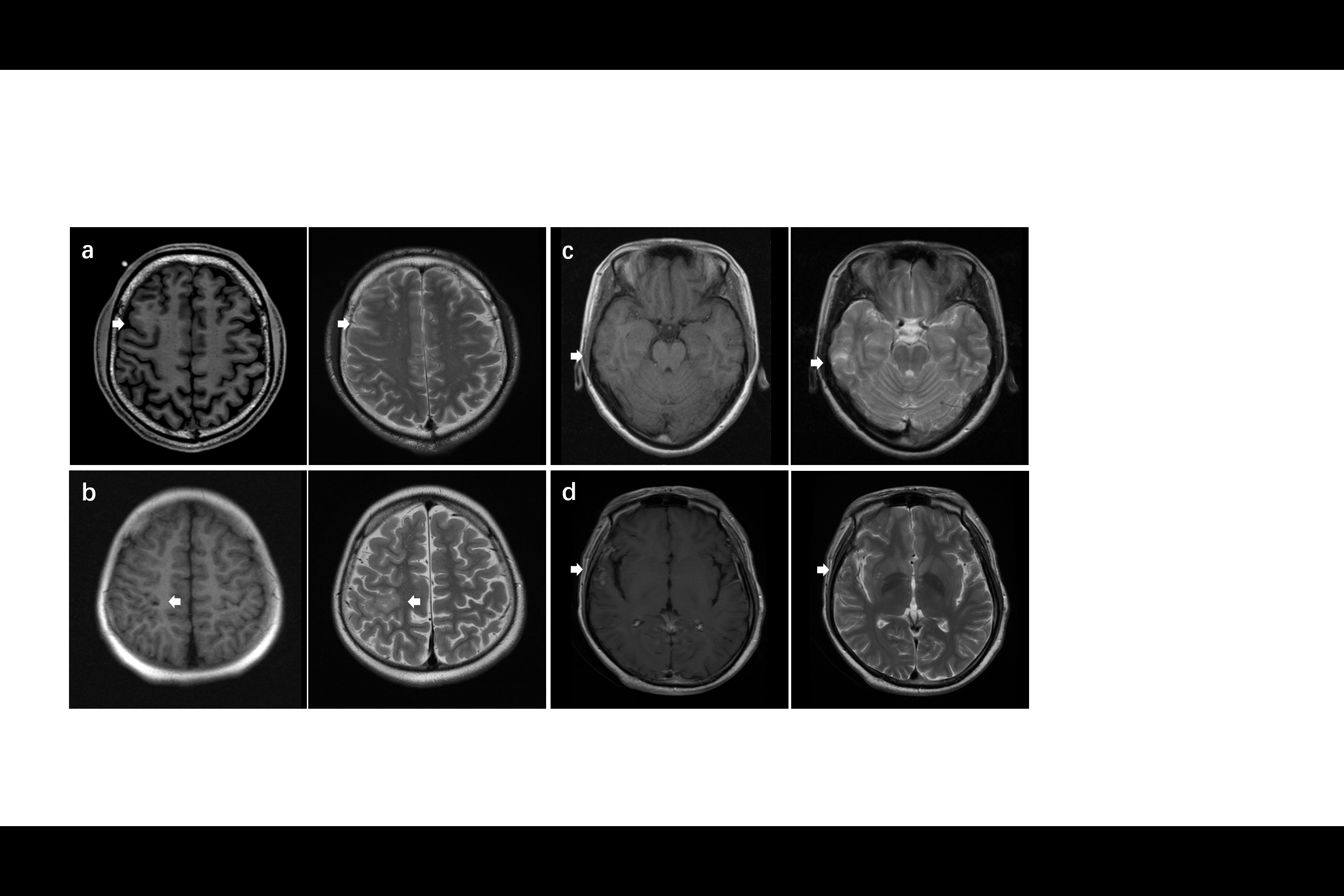


Supplementary figure 2. Examples of MRI for FCD and GNTs. a. Focal cortical thickening was presented in the right middle frontal gyrus, the pathology was FCD type Ic. b. Focal cortical thickening and increased signal on T2-weighted imaging in the right parietal lobe, the pathology was FCD type II. c. Focal hypointense on T1WI, and hyperintense on T2WI in the right temporal lobe, the pathology was DNTs. d. Focal hyperintense on T2WI and enhancement on T1WI in the right temporal lobe, the pathology was GGs. The white arrow pointed to the lesion. FCD: focal cortical dysplasia; GNTs: glioneuronal tumors; DNTs: dysembryoplasic neuroepithelial tumors; GGs: gangliogliomas.

**Supplementary figure 3** **The workflow of data preparation and machine-learning based modeling**


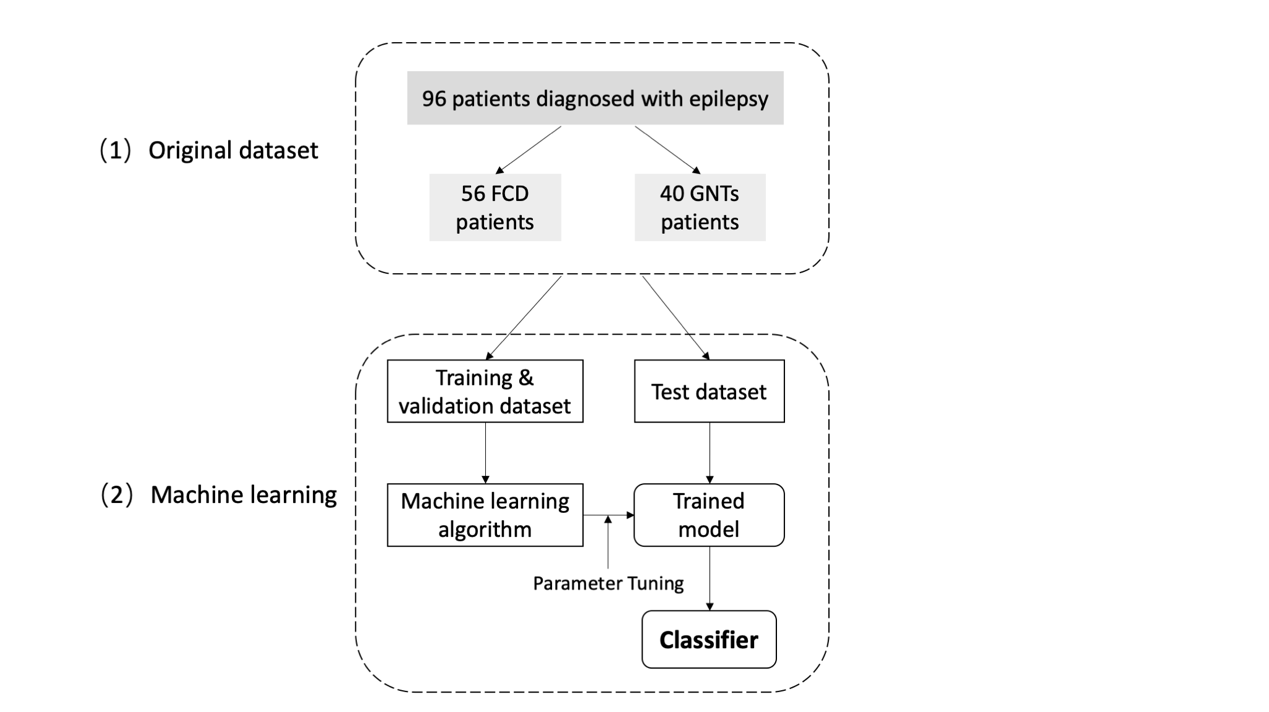


(1) Ninety-six epileptic patients were recruited in the original dataset, including 56 patients with FCD and 40 patients with GNTs. (2) The original dataset was split into a training & validation dataset and a test dataset. Seven different machine learning algorithms were selected for training based on the training and validation dataset. Trained models were achieved after parameter tuning. The final classifier was determined according to the comparison of each trained model's prediction performance.

**Supplementary table 1 Comparison of preoperative diagnostic accuracy between conventional method and random forest algorithm**

|  | Yes (n) | No (n) | Ratio |  |
| --- | --- | --- | --- | --- |
| the consistency between preoperative diagnosis and postoperative pathology | 73 | 23 | 76% | χ2=6.184  p=0.013 |
| the prediction accuracy of preoperative diagnosis by Random Forest | 86 | 10 | 89.6% |  |

Chi-Squared (χ2) Statistics was used for the comparison. * P < 0.05 was considered statistically significant.

**Supplementary table 2 Clinical characteristics of patients with FCD and GNTs（≤4years）**

| Variable | FCD  (n=24) | GNTs  (n=7) | Overall  (n-31) | P-value |
| --- | --- | --- | --- | --- |
|  |  |  |  |  |
| Course of disease(m) | 65±82 | 55±69 |  | 0.755 |
| MRI features, n(%) |  |  |  | 0.032 |
| Typical characteristics of GNTs | 1 (4.2%) | 5 (71.4%) | 6 (19.4%) |  |
| Typical characteristics of FCD | 17 (70.8%) | 2 (28.6%) | 19 (61.2%) |  |
| None | 6 (25%) | 0 (0%) | 6 (19.4%) |  |
| Number of AEDs, n(%) |  |  |  | 0.232 |
| None | 0 (0%) | 0 (0%) | 0(0%) |  |
| 1 drug | 2 (7.1%) | 3 (42.9%) | 5 (16.1%) |  |
| 2 drugs | 4 (14.2%) | 3 (42.9%) | 7 (22.6%) |  |
| ≧3 drugs | 18 (78.7%) | 1 (14.2%) | 19 (61.3%) |  |
| Scalp EEG Biomarkers of FCD, n(%) | | | | 0.111 |
| Negative | 3 (12.5%) | 5 (71.4%) | 8(25.8%) |  |
| Positive | 21 (87.5%) | 2 (28.6%) | 23(74.2%) |  |

Welch's t-test was used for course of disease(m). Chi-Squared (χ2) Statistics was used for other features. * P < 0.05 was considered statistically significant.

**Supplementary table 3 Clinical characteristics of patients with FCD and GNTs（>4years）**

| Variable | FCD  (n=32) | GNTs  N(n=33) | Overall  N(65) | P-value |
| --- | --- | --- | --- | --- |
|  |  |  |  |  |
| Course of disease(m) | 134±124 | 72±114 |  | 0.039 |
| MRI features, n(%) |  |  |  | <0.001^*^ |
| Typical characteristics of GNTs | 1 (3.1%) | 24 (72.7%) | 25 (38.5%) |  |
| Typical characteristics of FCD | 19 (59.4%) | 4 (12.1%) | 23 (35.4%) |  |
| None | 12 (37.5%) | 5 (15.2%) | 17 (26.1%) |  |
| Number of AEDs, n(%) |  |  |  | <0.001^*^ |
| None | 1 (3.1%) | 8 (24.2%) | 9 (13.9%) |  |
| 1 drug | 1 (3.1%) | 10 (30.3%) | 11 (16.9%) |  |
| 2 drugs | 11 (34.3%) | 10 (30.3%) | 21 (32.3%) |  |
| ≧3 drugs | 19 (59.5%) | 5 (15.2%) | 24 (36.9%) |  |
| Scalp EEG Biomarkers of FCD, n(%) | | | | 0.498 |
| Negative | 18 (56.3%) | 22 (66.7%) | 40 (61.5%) |  |
| Positive | 14 (43.7%) | 11 (33.3%) | 25 (38.5%) |  |

Welch's t-test was used for course of disease(m). Chi-Squared (χ2) Statistics was used for other features. * P < 0.05 was considered statistically significant.
